# Brain damages in ketamine addicts as revealed by magnetic resonance imaging

**DOI:** 10.3389/fnana.2013.00023

**Published:** 2013-07-17

**Authors:** Chunmei Wang, Dong Zheng, Jie Xu, Waiping Lam, D. T. Yew

**Affiliations:** ^1^Brain Research Center, Institute of Chinese Medicine, The Chinese University of Hong KongHong Kong SAR, China; ^2^Department of Neurology, Guangzhou Brain Hospital, Affiliated Hospital of Guangzhou Medical UniversityGuangzhou, China; ^3^Department of Anatomy, Zhongshan School of Medicine, Sun Yat-Sen UniversityGuangzhou, China; ^4^Faculty of Medicine, Brain Research Center, School of Biomedical Sciences, The Chinese University of Hong KongHong Kong SAR, China

**Keywords:** ketamine, addiction, brain, lesion, atrophy, magnetic resonance imaging (MRI)

## Abstract

Ketamine, a known antagonist of N-methyl-D-aspartic (NMDA) glutamate receptors, had been used as an anesthetic particularly for pediatric or for cardiac patients. Unfortunately, ketamine has become an abusive drug in many parts of the world while chronic and prolonged usage led to damages of many organs including the brain. However, no studies on possible damages in the brains induced by chronic ketamine abuse have been documented in the human via neuroimaging. This paper described for the first time via employing magnetic resonance imaging (MRI) the changes in ketamine addicts of 0.5–12 years and illustrated the possible brain regions susceptible to ketamine abuse. Twenty-one ketamine addicts were recruited and the results showed that the lesions in the brains of ketamine addicts were located in many regions which appeared 2–4 years after ketamine addiction. Cortical atrophy was usually evident in the frontal, parietal or occipital cortices of addicts. Such study confirmed that many brain regions in the human were susceptible to chronic ketamine injury and presented a diffuse effect of ketamine on the brain which might differ from other central nervous system (CNS) drugs, such as cocaine, heroin, and methamphetamine.

## Introduction

Ketamine, a known antagonist of N-methyl-D-aspartic (NMDA) glutamate receptors, had been used as an anesthetic, particularly for pediatric or for cardiac patients. Ketamine employed in prescribed medical conditions had its advantages as it did not increase intracranial pressure during neurosurgery (Schmittner et al., 2007) and had no postoperative neurological damage when used in cardiopulmonary bypass patients (Smith et al., 2006). Medically, ketamine has also been proposed for anticonvulsive control (Dickenson and Ghandehari, 2007) and for controlling injury after stroke via it action on the glycine, zinc, and magnesium components of the glutamate binding sites (Collins et al., 1989); thus, protecting neuronal loss after stroke (Meldrum et al., 1987) and preventing of the spread of depolarization after injury (Hertle et al., 2012). This drug is, however, not without flaw. Being one of the noncompetitive NMDA receptor antagonists like phencyclidine and MK801, it would produce schizophrenia like psychosis in human (Dickerson and Sharp, 2006). Unfortunately, ketamine has now become an abusive drug in many parts of the world and chronic and prolonged usage led to damages of many organs in experimental animals (Yeung et al., 2009; Chan et al., 2011; Tan et al., 2011a; Wai et al., 2012; Wong et al., 2012). The damage on the nervous system included neuronal loss, synaptic changes, changes in functional magnetic resonance imaging (fMRI) activities, and the formation of mutated tau protein in neurons as described in models of rodents and monkeys (Yeung et al., 2010a; Sun et al., 2011; Yu et al., 2012).

In the higher primate, chronic treatment of ketamine induced changes of apoptotic markers in the prefrontal cortex and abnormal behavior in movement, walking, jumping, and climbing (Sun et al., 2012). fMRI in monkeys also revealed hyperactivity in entorhinal cortex, striatum regions, but hypoactivity in midbrain and visual cortex (Yu et al., 2012). In the mice, prefrontal hippocampal damages (Yeung et al., 2010a; Wai et al., 2013), pain altercations, and schizophrenic like behavior (Becker et al., 2003, 2006) had been documented and GABA receptor and changes of dopaminergic neurons were recorded in mouse model (Tan et al., 2011b, 2012). In the human brain, so far, few studies appeared in the literature. Our group, for example, revealed fMRI hypoactivities in the cerebellum of addicts (Chan et al., 2012). Narendran et al. (2005) reported that ketamine addicts exhibited selective up-regulation of dopamine D1 receptor via biochemistry. No study was ever put forward summarizing human central nervous system (CNS) lesions as yet. This paper described for the first time via magnetic resonance imaging (MRI) changes in the addicts of 0.5–12 years of ketamine addiction, and demarcated the possible brain regions susceptible to ketamine damages.

## Materials and methods

### Subjects

This study had consents from patients and was approved by the ethical committee of Sun Yat-sen University, Guang Zhou, China. Twenty-one human ketamine addicts were employed in the study. The ages of these patients were between 19 and 48 years old, with only two above 31 (one of 38 and another of 48). They all had no previous medical history of brain trauma or neurological diseases. The dosage used by the patients was from 0.2 to 3 g a day but majority dosage was 1 g a day. Among them, 19 of these patients took ketamine daily, while only two took it twice or three times a week. The durations of drug addiction ranged from 0.5 to 12 years. The break down of addicts with three years addiction or below was *n* = 6. Four to six years addiction was *n* = 7. Seven years of addiction was *n* = 3 and over ten years of addiction was *n* = 5. The patient data were indicated in Table1. The brains of these patients were subjected to MRI image. Three age matched normal subjects (ages 19, 21, and 40 years old) were used as control for MRI imaging.

**Table 1:** **Characteristics of the ketamine addicts**.

**Years of addiction**	**No. of addict**	**Dosage**	**Frequency**	**Drug manner**
0.5	1	0.2 g	Twice a week^Δ^	Nasal absorption
1	2	>0.5 g	Every day or three times a week^ΔΔ^	Nasal absorption
2	1	>0.5 g	Every day	Nasal absorption
3	2	>0.5 g	Every day	Nasal absorption
4	2	≥1 g	Every day	Nasal absorption
5	2	≥1 g	Every day	Nasal absorption
6	3	≥1 g	Every day	Nasal absorption
7	3	≥1 g^*^	Every day	Nasal absorption
10	3	≥1 g	Every day	Nasal absorption
12	2	≥1 g	Every day	Nasal absorption

* *One of three patients took 3 g a day*.

Δ *The patient took ketamine along with amphetamine and ecstasy*.

ΔΔ *One patient took ketamine daily, another took ketamine three times a week*.

### Neuroimaging study

MRI was performed with a 3.0-Tesla imager (Achieva; Philips Medical Systems, Best, the Netherlands). The images were obtained with 5.0 mm section thickness (ST). The field of view (FOV) was 23 × 18 cm^2^ with 8 channel SENSE head coil. T1-weighted images were obtained with 2000 ms repetition time (TR) and 20 ms echo time (TE). T2-weighted images were obtained with TR and TE at 3000 ms and 20 ms and fluid attenuated inversion recovery (FLAIR) images were obtained at 11,000 ms TR and 20 ms TE. The total acquisition time for the sequences was about 30 min.

## Results

**Figure 1:**
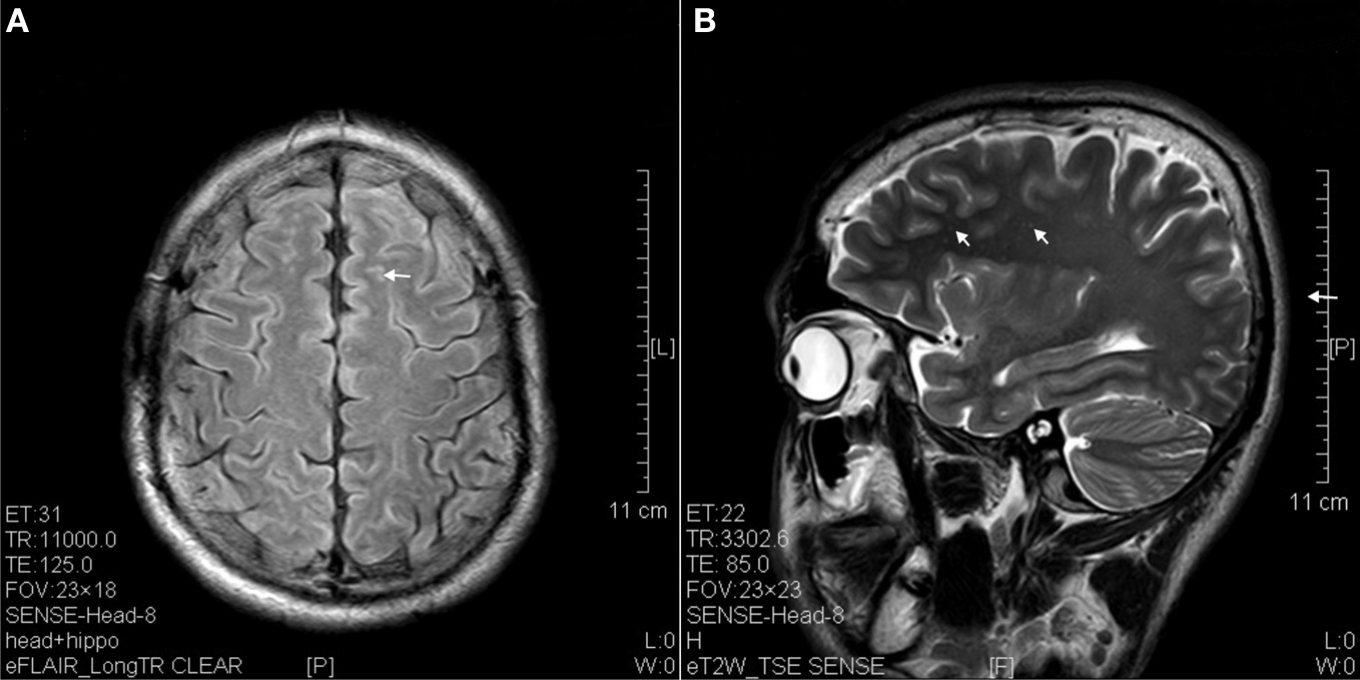
**Hyperintense spots (arrow) in superficial white matter and internal capsule of ketamine addicts. (A)** FLAIR imaging of a 1 year ketamine addict. **(B)** T2 imaging of a 3 years ketamine addict.

**Figure 2:**
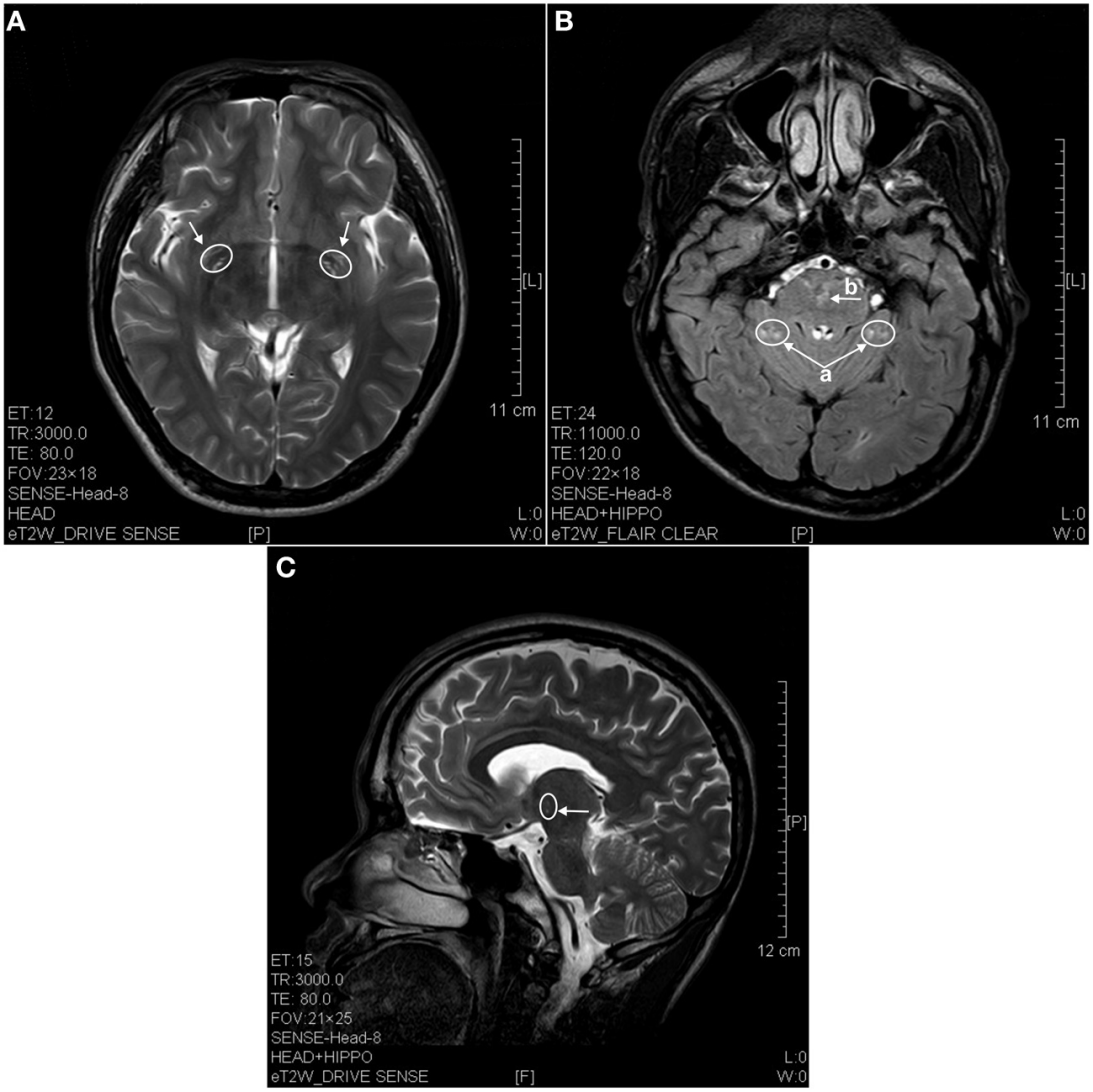
**MRI images of the brains of a 4 years ketamine addicts. (A)** T2 image of a horizontal brain section showing degenerative hyperintense spots in basal forebrain (arrow). **(B)** T2 image of a horizontal section showing hyperintense degeneration in cerebellum (a) and in pons (b). **(C)** T2 image of a sagittal section showing degeneration spots in diencephalon (thalamus).

**Figure 3:**
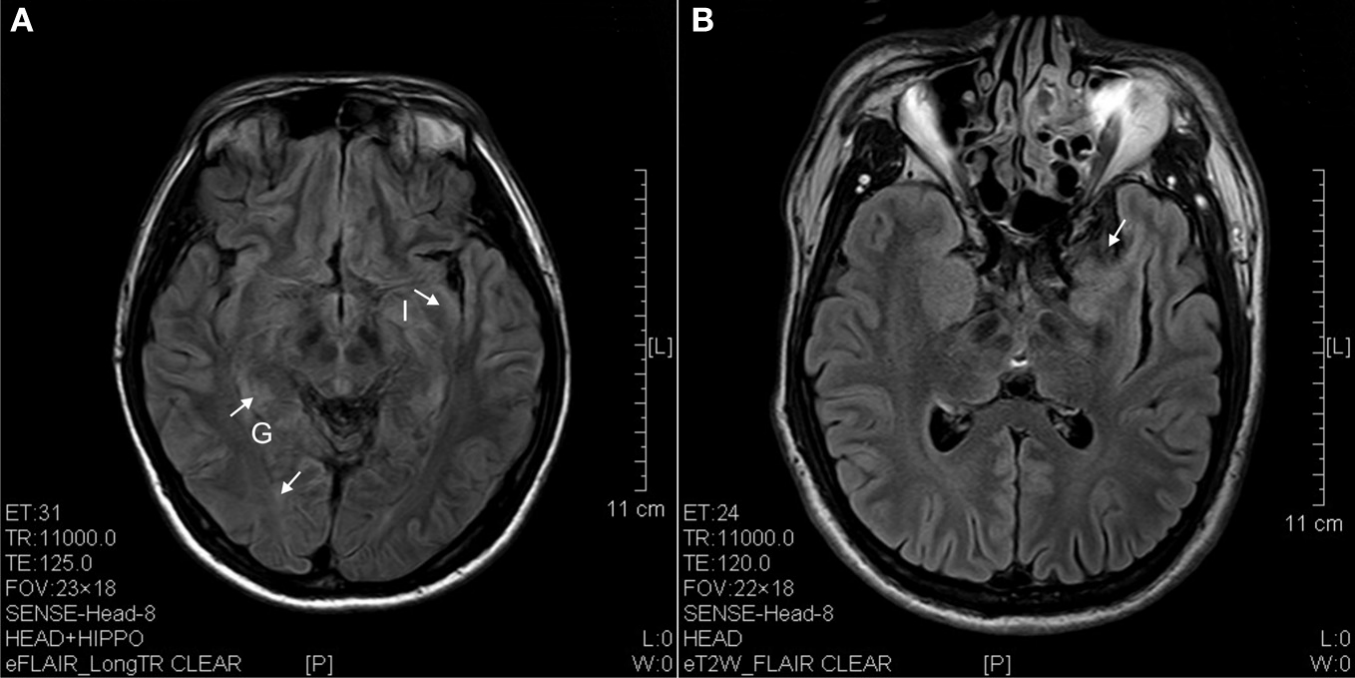
**FLAIR image of diffusion blockage as hyperintense spots in the parahippocampal gyrus (G) and insula (I) as well as atrophy of uncus (arrow). (A)** Ketamine addict of 4 years. **(B)** Ketamine addict of 5 years.

**Figure 4:**
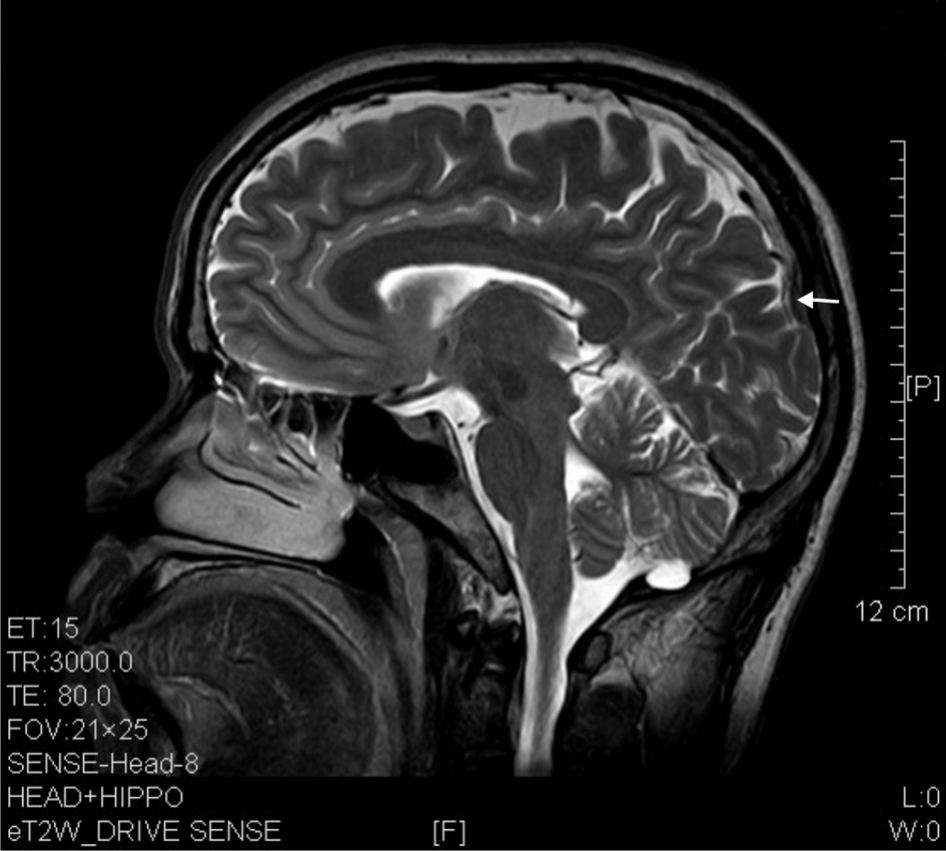
**T2 image showed parietal atrophy (arrow) in a sagittal brain section of a ketamine addict of 4 years**.

**Figure 5:**
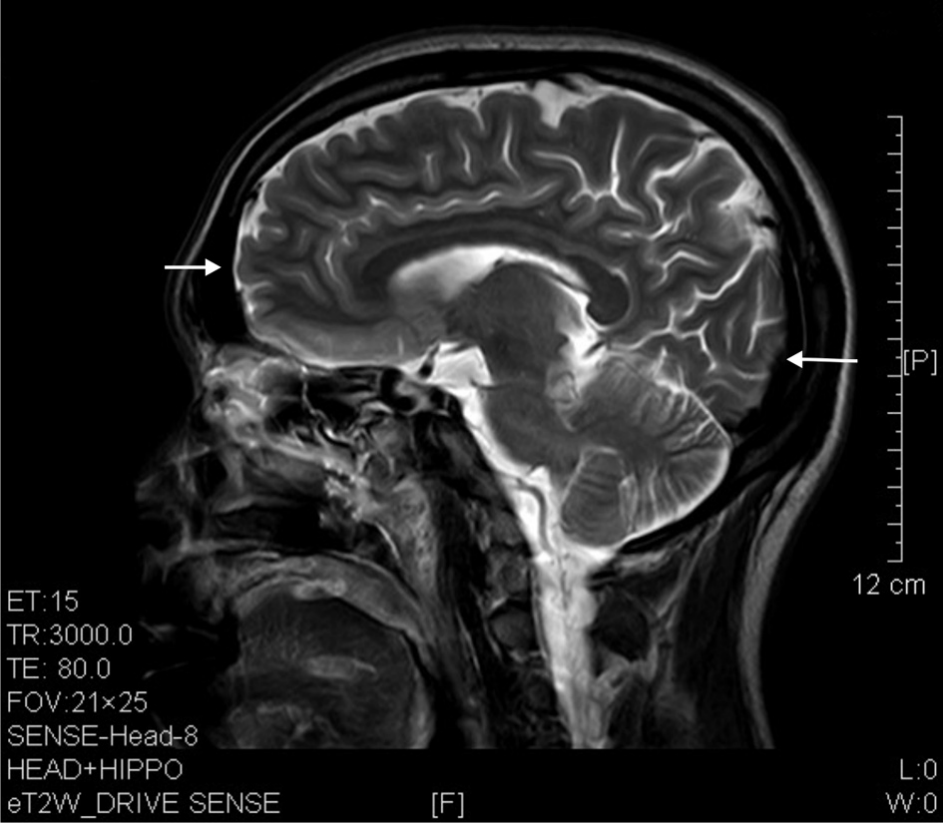
**T2 image showed prefrontal and occipital atrophy (arrows) in a sagittal brain section of a 7 years' ketamine addict**.

**Figure 6:**
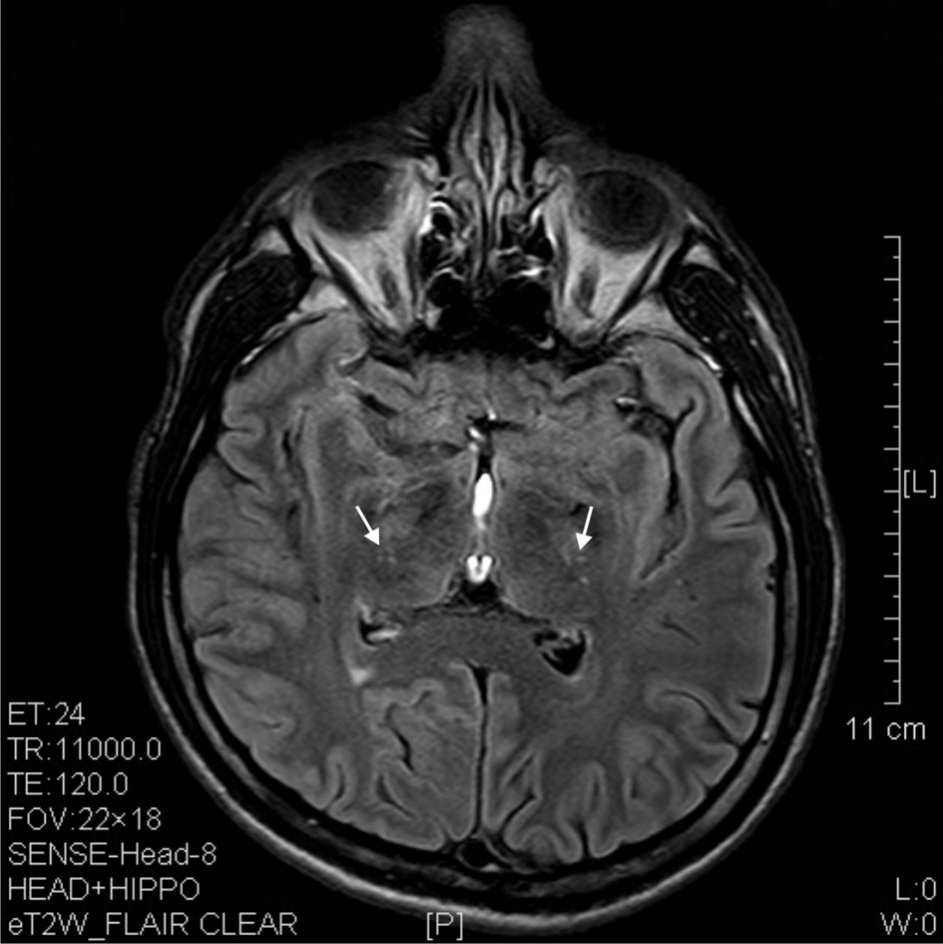
**T2 image showed hypertensive degenerative spots in corpus striatum (arrows) of a 6 years' ketamine addicts**.

**Figure 7:**
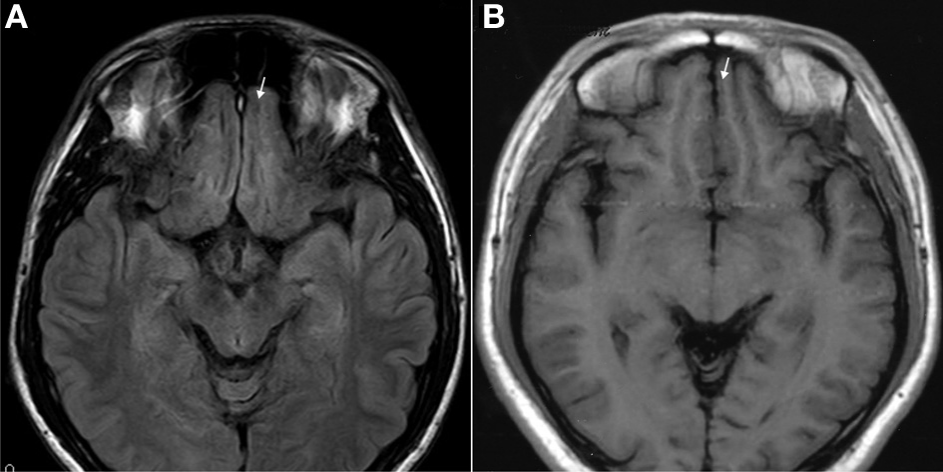
**T1 images showed atrophic basal prefrontal (gyrus rectus) lesion of a 0.5 years ketamine addicts who took three drugs including ketamine (A). (B)** Control with no retraction of gyrus rectus (arrow).

**Figure 8:**
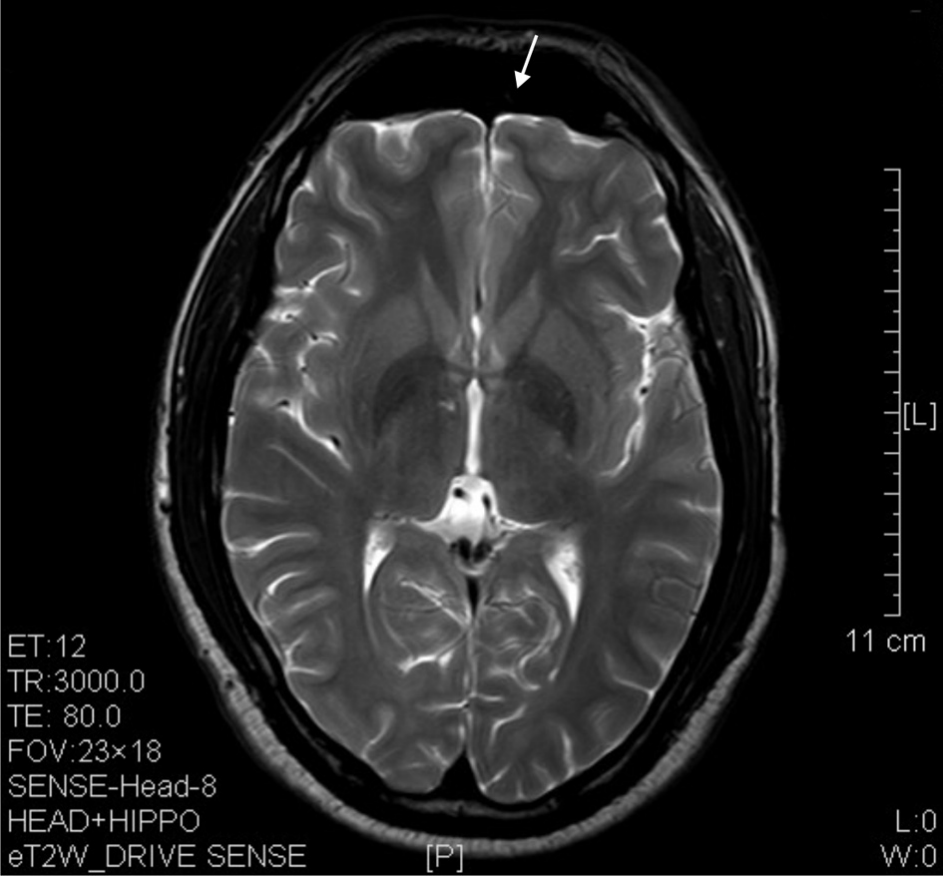
**T2 image showed significant prefrontal atrophy (arrow) in a horizontal brain section of a ketamine addict who had high dose of ketamine (3 g per day) for only 3 years**.

**Figure 9:**
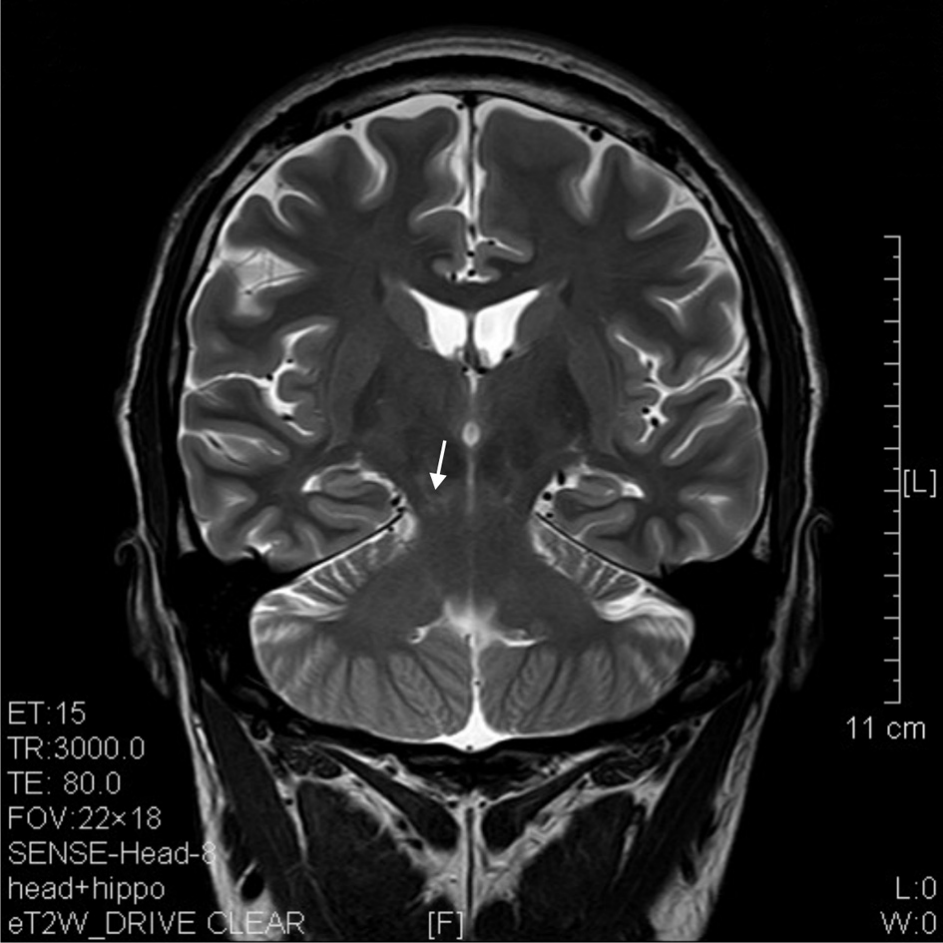
**T2 image of a coronal section that showed degenerative lesion (arrow) in the brainstem (midbrain) of a ketamine addict of 7 years**.

The results of lesions observed in all the 21 ketamine addicts were depicted in Table 2. Those who had two or less regions in the brain with lesions were classified as light damage. Those that had three to four regions in the brain with lesions were classified as moderate damage, and those with five or more regions with lesions were classified as severe damage. The MRI lesions initially were observed as hyperintense spots (holes or patches) of degeneration in the superficial white matter of the cortex which appeared as early as 1 year after ketamine addiction (Figure 1A), while each lesions spread to the internal capsule by 3 years of addiction (Figure 1B). Slightly after, patches of hyperintense degeneration spots appeared in the basal forebrain (Figure 2A), cerebellum, and pons (Figure 2B), and diencephalon at 4 years of addiction (Figure 2C). Likewise, diffusion blockage was illustrated by FLAIR image in the parahippocampal gyrus and insula, also by 4 years of addiction (Figure 3A), while atrophy of the parahippocampal gyrus was observed a bit later by 5 years of addiction (Figure 3B). Atrophy of the other parts of cortex was first noted after 4 years of addiction, usually with atrophy on only a small region of the cortex (Figure 4) and extended to two or three regions (usually frontal, parietal, and occipital) of the cortex by 7 years of addiction (Figure 5). Hyperintense lesions were also observed in the corpus striatum by 6 years (Figure 6). In this patient cohort, one patient had a combination of drugs and was taking ketamine together with amphetamine and ecstasy. He demonstrated early atrophy of cortex after taking the three drugs together in 0.5 years, in which the basal prefrontal gyrus rectus already exhibited significant atrophy (Figure 7A) when compared with control (Figure7B). Similarly, cortical atrophy also occurred early in another patient who had used a high dose of ketamine, in this case 3 g per day for 3 years (Figure 8). After 7 years of addiction, in all other patients, lesions then appeared in the midbrain (Figure 9). From 10 to 12 years of addiction, all lesion sites were as those described above.

**Table 2:** **The summary of the lesions and atrophy in the brains of ketamine addicts in term of years of addiction**.

**Years of abuse**	**Cerebellum or cerebella**	**Holes/degenerative patches in white matter**	**Cortex**	**Limbic system (Uncus or entorhinal)**	**Internal capsule**	**Capsule striatum**	**Diencephalon**	**Brainstem**	**Atrophy of cortex (frontal/partial/occipital)**	**Severity of brain damage#**
0.5	−	+	+^Δ^	−	−	−	−	−	+	Light
1	+	+	−	−	+	−	−	−	−	Light
2	+	+	−	−	+	−	−	−	+	Moderate
3	+	+	+^ΔΔ^	−	+	−	−	−	+	Moderate
4	+	+	+	−	+	−	+	+^*^	+	Severe
5	+	+	+	+	+	−	+	+	+	Severe
6	+	+	+	+	+	+	+	+	+	Severe
7	+	+	+	+	+	+	+	+^**^	+	Severe
10	+	+	+	+	+	+	+	+	+	Severe
12	+	+	+	+	+	+	+	+	+	Severe

*+, stands for the positive lesion; −, stands for the negative lesion*;

Δ This is a patient on three types of abusive drug including ketamine and who had early lesions;

ΔΔ This is a patient that had 3 g per day of ketamine;

* *Lesion firstly appeared in pons by 4 years of addiction*.

** Lesion in the midbrain appeared by 7 years of addiction;

*#Light damage meants two or less regions affected in the brain, moderate indicates three to four brain regions affected, and severe meants five or more brain regions affected*.

Medical histories indicated that addicts over 4 year of ketamine addiction displayed memory deficits and anxiety depression while those from the 5 to 7 years developed definitive ataxia and by 7 years and above, dyskinesia became obvious.

## Discussion

This study revealed the lesions in many regions of the brain of ketamine addicts. These lesions appeared as minute patches in the first year and became larger sites of atrophy by 4 years of addiction. The majority of the addicts was on dosage of 1 g per day and used ketamine daily for several years. In this work, since the volunteers were mostly below 30 years old and only 2 individuals above 30 years old, a comparison of the effect of age upon addiction was not conclusive in this stage, even though we had seen no worse in the aged group (above 30 years old) when compared with the slightly younger old. A study of age response would definitely be conducted in future. However, it is well-known that ketamine addicts were usually young as represented in this cohort. The brain regions affected were prefrontal, parietal, occipital, limbic, brainstem, and corpus striatum. The lesions affected both the gray and white matter, i.e., neurons and nerve fibers in the human; these were similar to those reported earlier by us in the mice and the monkey (Yu et al., 2012). This MRI study also collated with the work by Morgan and Curran suggesting a loss of memory via psychological examination in chronic ketamine abuses (Morgan and Curran, 2006). In animals, prefrontal cortex apoptosis, mutated tau aggregation, brainstem chemical changes, and cerebellar apoptosis had been reported (Mak et al., 2010; Yeung et al., 2010b; Sun et al., 2011, 2012; Tan et al., 2011b, 2012; Yu et al., 2012; Wai et al., 2013). In fact, in the mice model, it had been documented both neurons and fibers (white matter) were both targets like in this report consisting of human subjects (Mak et al., 2010; Yeung et al., 2010b). Along with structural changes, fMRI and functional studies confirmed functional and cognitive derangements (Morgan and Curran, 2006; Sun et al., 2011, 2012; Chan et al., 2012; Yu et al., 2012). This human MRI brain imaging on the ketamine addicts thus consolidated that the areas of lesion in mice, monkey, and human were essentially similar. We now have clear and unequivocal proof of damages in the CNS upon chronic use of ketamine in human. As for the combination of drugs, we had only one individual on three types of drug—amphetamine, ecstasy and ketamine. The individual was only on such drugs for half a year with low dosage of ketamine, but his lesions were more vigorous than the addicts who had been on the drug for 3 years. This preliminary observation suggested severe detrimental effects on brain upon the combination of abusive drugs in the addicts.

Hyperdensity spots in computerized axial tomography (CAT) scan usually pointed to demyelination or metabolic changes (Brismar and Ozand, 1994; Matsushima et al., 1997). For MRI, while hyperintensive spot in T1 imaging pointed to toxicity or metabolic lesion, T2 hyperintensive could refer to demyelination and hardening of arteries (Hyttinen et al., 2009). Other studies on T1, T2, and FLAIR MRI indicated even more possibilities for hyperintensive spotty or non-spotty lesions (Cakirer et al., 2003). For instance, T1 hyperintensity could be related to infarct, infection, axonal damage, hemorrhage or vascular change and neoplasm (Cakirer et al., 2003), T2 hyperintensite image might indicate cytotoxicity, vasogenic edema whilst metabolic toxicity, encephalopathy, vasogenic edema would restrict diffusion and contributed to FLAIR hyperintensity (Cakirer et al., 2003). In our patients, these lesions observed were probably related to cytotoxicity, axonal damage, and vasogenic edema which we indicated as “degeneration” while atrophy of cortex was the last sequel of the events.

There were some studies on the neuroimaging of addicts using different drugs. Reneman et al. (2006), for example, using proton magnetic resonance spectroscopy (1H-MRS) suggested the cortical and subcortical reduction of serotonin transporter in ecstasy patients. Also using magnetic resonance spectroscopy, de Win et al. (2008a) reported thalamic damage by ecstasy. Apart from the thalamus, other subcortical structures in the globus pallidus and putamen were also affected by esctacy (de Win et al., 2008b). Using simple MRI and diffusion tensor imaging (DTI) techniques, other areas of the brain were reported to be damaged by ecstasy, e.g., the limbic cortex (Thompson et al., 2004), the medial temporal cortex (Thompson et al., 2004), callosum (Salo et al., 2009), limbic system (Sowell et al., 2010), and hippocampus (Den Hollander et al., 2012). Front-cortical and striatal damages were featured in the cocaine treated rat by MRI (Gozzi et al., 2011), callosum damage by DTI in human cocaine addicts (Moeller et al., 2007) and damage in frontal white matter in prenatally cocaine exposed human addicts (Smith et al., 2001). In the heroin addicts, for example, decreased gray matter was observed in the frontal, cingulate, and occipital cortices (Wang et al., 2012). In the cocaine addicts, cue-induced cocaine crowing involved the left dorsolateral frontal cortex and the anterior cingulate (Bolla et al., 1998; Maas et al., 1998; Wexler et al., 2001; Goldstein et al., 2004). These areas were also found to be involved in methamphetamine craving (Yin et al., 2012). Toda revisited these damaged areas in a review and proposed that these areas had glutamatergic projections to the nucleus accumbens (Toda, 2012). In this study, the ketamine damaged regions in the human subjects were put forward for the first time and resolved that they included diffusely many regions: frontal, parietal, occipital cortices, parahippocampal gyrus, striatum (including caudate), cerebellum, and brainstem. However, no obvious involvement was observed in the cingulate gyrus. Some of these areas were the same as cocaine and ecstasy patient, e.g., frontal, striatum, and limbic. However, ketamine presented a rather diffuse effect in many other of areas of the brain which might differ from cocaine, heroin, or methamphetamine.

This piece of work further suggests that addicts even on a single drug alone like ketamine might lead to atrophy of the brain after a few years of addiction. As addicts usually take ketamine at different time and ad lib, it was very difficult to compare dosages. In this work, there were hints that increasing dosage or combination of ketamine with other abusive drug would hasten the damages. It is important therefore to compare the years of duration and with a certain level of dosage which at least led some usable data. Further work should emphasize on the comparison of the interaction of these abuse drugs.

### Conflict of interest statement

The authors declare that the research was conducted in the absence of any commercial or financial relationships that could be construed as a potential conflict of interest.
